# The Prejudice towards People with Mental Illness (PPMI) scale: structure and validity

**DOI:** 10.1186/s12888-018-1871-z

**Published:** 2018-09-12

**Authors:** Amanda Kenny, Boris Bizumic, Kathleen M. Griffiths

**Affiliations:** Research School of Psychology, The Australian National University, Building 39 Science Road, Canberra, ACT 2601 Australia

**Keywords:** Prejudice, Mental illness, Attitudes, Stigma, Scale development

## Abstract

**Background:**

Although there is a substantial body of research on the stigma associated with mental illness, much of the extant research has not explicitly focused on the concept of prejudice, which drives discriminatory behaviour. Further, research that has investigated prejudice towards people with mental illness has conceptual, theoretical and psychometric limitations. To address these shortcomings, we sought to develop a new measure, the Prejudice towards People with Mental Illness (PPMI) scale, based on an improved conceptualisation and integration of the stigma and prejudice areas of research.

**Methods:**

In developing the new scale, we undertook a thematic analysis of existing conceptualisations and measures to identify a pool of potential items for the scale which were subsequently assessed for fidelity and content validity by expert raters. We tested the structure, reliability, and validity of the scale across three studies (Study 1 *N* = 301; Study 2 *N* = 164; Study 3 *N* = 495) using exploratory factor, confirmatory factor, correlational, multiple regression, and ordinal logistic regression analyses using both select and general community samples.

**Results:**

Study 1 identified four factors underlying prejudice towards people with mental illness: fear/avoidance, malevolence, authoritarianism, and unpredictability. It also confirmed the nomological network, that is, the links of these attitudes with the proposed theoretical antecedents and consequences. Studies 2 and 3 further supported the factor structure of the measure, and provided additional evidence for the nomological network.

**Conclusions:**

We argue that research into prejudice towards people with mental illness will benefit from the new measure and theoretical framework.

**Electronic supplementary material:**

The online version of this article (10.1186/s12888-018-1871-z) contains supplementary material, which is available to authorized users.

## Background

Researchers have widely studied mental illness (MI) stigma because it has detrimental effects on people with MI, such as widespread discrimination, exacerbated symptoms, and poor treatment outcomes (e.g., [1]). The concept of stigma includes multiple components, such as stereotypes, prejudice, and discrimination [2]. Within the literature its use has been primarily confined to studies of illness. By contrast, researchers who study prejudice see it as a specific negative attitude, which has largely been studied in relation to ethnic and racial outgroups [3]. The concept of prejudice itself has rarely been the explicit focus of studies involving MI, and many scales measuring stigma do not explicitly focus on, or in some cases do not include items measuring, prejudice [1]. Recently, Phelan et al. wrote: “the strong congeniality and large degree of overlap we found between models of stigma and prejudice should encourage scholars to reach across stigma/prejudice lines when searching for theory, methods and empirical findings to guide their new endeavors” ([3], p. 365) We will follow these researchers’ call, and while reviewing the broad literature on stigma, focus on the construct of prejudice towards MI and its measurement.

For the purpose of this investigation, we will use a definition of prejudice as a negative outgroup attitude [4], and an attitude as a positive or negative evaluation of an object [5]. These definitions are widely, though not universally, endorsed. According to this conceptualisation, attitudes are distinct from stereotypes, which involve generalisations about group members [6], and can be known without being endorsed. We therefore see prejudice as the central component of stigma that drives behaviour (discrimination), and the avenue with the most potential to modify the detrimental effect of these attitudes. Thus, in integrating the literature on stigma and prejudice, we will focus primarily on the concept of prejudice, its measurement, and its theoretical causes and consequences, building on both stigma and prejudice literature, and will not focus on the related concepts of stereotypes and discrimination.

### Problems in the study of prejudice towards people with MI

Researchers have developed numerous scales to assess stigma towards people with MI, and many of these scales include items measuring prejudice [1]. The scales in this area, however, often have conceptual and psychometric problems (see [1] for a recent comprehensive summary of problems of existing stigma measures, including its psychometric properties; these researchers note that even 2/3 of all published measures of stigma have not had any psychometric evaluations, and most of those that have had such evaluations still have numerous problems.). Researchers in the field of psychological measurement have emphasised the importance of construct validity and the related nomological network, which describes the theoretical antecedents and consequences of a psychological construct [7]. Construct validity should be a central aspect of scale development, but measures in this area often do not elucidate the nomological network (e.g., [8–11]).

Many scales in the area fail to define what they measure and focus on diverse beliefs, opinions, attitudes, and stereotypes related to MI (e.g., [12–14]). This becomes important as people can be aware of stereotypes without endorsing them, and even scales designed to measure attitudes often include items presenting non-evaluative opinions (e.g., [15, 16]). Many measures do not specify the construct and their items have various targets, such as the person, MI, or treatment (e.g., [8, 14, 17–19]), which often do not contain the evaluative component central to an attitude [5] and may not be linked to behaviour and therefore influence discrimination. Construct validity is central to showing that a measure reflects the true theoretical meaning of a concept.

Although studies indicate that attitudes towards people with MI are multidimensional, and commonly found dimensions relate to avoidance, exclusion, fear, benevolence, and authoritarian control [20, 21], there is no agreement on the number and nature of dimensions. Moreover, scales often lack a replicable factor structure. For example, the factor structures of widely-used measures of attitudes towards people with MI, the Opinions about Mental Illness (OMI) scale [22], and the Community Attitudes toward the Mentally Ill scale (CAMI) [14] have not been replicated (e.g., for OMI [23, 24]; for CAMI [20, 25]). Without a clear and replicable factor structure, evaluating any variation in attitudes over time or as a result of interventions is fraught with a lack of clarity around the mechanisms of change and influence.

On the most fundamental level, even widely-used scales, such as the OMI and CAMI, contain double-barrelled items, which are items that include two separate ideas (e.g., [13, 18, 26]). Including such items is poor psychometric practice because participants may be responding to either of two ideas. In addition, scales often fail to address acquiescent response bias (e.g., [13, 26, 27]). To reduce acquiescence, researchers construct balanced scales with equal numbers of positively-keyed and negatively-keyed items [28]. Furthermore, researchers often overlook social desirability factors when measuring negative attitudes towards people with MI [2, 29].

Researchers today are not able to specifically study prejudice, as most measures are focused on a diversity of phenomena, and therefore may miss the central attitudinal aspect of prejudice that drives discriminatory behaviours. If a diverse set of phenomena are included in a measure, then this hinders the development of our understanding of the phenomenon, including its nomological network.

It should be noted that problems that affect the study of prejudice towards people with MI could perhaps be ameliorated by including a validated measure of generalised prejudice and applying it to people with MI. There, however, does not appear to exist a well-validated and multidimensional measure of prejudice that could be applied to people with MI. We were, for example, able to find only one-item feeling thermometer measures, typically used to measure attitudes towards ethnic groups, being applied to people with MI (e.g., [30, 31]), but these measures cannot capture the multidimensional nature of prejudice against people with MI. A problem in the area of social psychological study of prejudice is that measures of prejudice are often created ad hoc, and even when they are not created ad hoc, they are often aimed at specific groups, such as specific ethnic minorities, and therefore may not be appropriate when applied to people with MI. Accordingly, a measure that specifically targets prejudice against people with MI is needed.

### Nomological network

To inform the nomological network, we argue that prejudice towards people with a MI represents multidimensional negative attitudes towards people with MI. In addition, we argue that extensive literature from prejudice research provides a useful integration of existing fields of research into the study of negative attitudes towards people with MI. Based on the extensive research literature on prejudice, we posit the following antecedents and consequences.

#### Antecedents

Social dominance orientation (SDO) and right-wing authoritarianism (RWA) are ideological beliefs that predispose people to prejudice towards many groups [32]. SDO, as an orientation towards non-egalitarianism and preference for group-based dominance [33], predicts prejudice towards groups perceived inferior [34], and may relate to less benevolent and sympathetic attitudes towards people with MI. RWA reflects traditional, conservative, and authoritarian social attitudes, and relates to prejudice against threatening groups [30], and conceivably relates to fearfulness and authoritarian control of people with MI. RWA and SDO have been shown to be the two most important predictors of generalised prejudice, and together they explain most of its variance [35].

Research has also shown that lack of empathy predicts generalised prejudice over and above the influence of SDO and RWA [36], and may therefore relate to less benevolence towards people with MI. In addition, a meta-analysis has linked the personality traits of lower agreeableness and openness to experience to generalised prejudice and these appeared to be most important effects of personality traits on prejudice [32]. It could be expected that lower agreeableness conceivably relating to less benevolence, and lower openness to avoidance of people with MI. Finally, less contact with people with MI is associated with more negative attitudes [37], possibly predisposing people to fear and avoidance of people with MI. Contact itself has been used as a frequent cause of stigma towards people with MI, whereas RWA, SDO, empathy, and personality traits were not. Nonetheless, all these concepts have been shown to be highly important theoretical underpinnings of prejudice in general, and therefore, should be comprehensive underpinnings of prejudice against people with MI.

#### Consequences

The two main consequences of attitudes towards people with MI would be attitudes towards people with specific mental illnesses and discriminatory behaviours. Accordingly, general prejudice towards people with MI should translate into people’s attitudes towards people with specific mental illnesses. Research has identified two dimensions underlying social perception: warmth and competence, which translate into disliking (low warmth) and disrespect (low competence) [38]. General prejudice towards people with MI may predispose people to dislike and/or disrespect people with a specific MI, such as schizophrenia or depression. Thus, although there may be differences in liking and respect towards people with specific mental illnesses, these attitudes are expected to be influenced by general attitudes towards people with MI. Additionally, prejudice may lead to discrimination towards people with MI [2] because attitudes have been found to be consistently linked to behavioural outcomes [39].

### Study aims

This project aimed to address limitations of research into prejudice towards people with MI, and develop a scale – the Prejudice towards People with a Mental Illness (PPMI) scale – to measure this prejudice. To this end, in constructing the PPMI scale we (i) limited the attitude object to *people* with MI; (ii) defined prejudice as negative attitudes, and excluded other components of stigma; (iii) aimed to develop a balanced scale; and (iv) measured social desirability to control for response biases. The items of the PPMI scale were developed to address topics identified in a thematic analysis of existing measures. The factor validity of the PPMI scale was assessed using an exploratory and confirmatory factor analyses. Based on past research we predicted that:

- **Hypothesis 1:** Prejudice associated with MI is multidimensional.

Construct validity was measured by exploring relationships between the PPMI scale and antecedents and consequences. In particular, based on past research we predicted that:

- **Hypothesis 2:** The prejudice towards people with MI as measured by the PPMI scale relates to antecedents of higher RWA and SDO, and lower agreeableness, openness to experience, empathy, and contact.
- **Hypothesis 3:** The consequences of MI prejudice are disliking and disrespect for people with specific MI and discriminatory behaviours.

We anticipated that the precise relationships of the antecedents and consequences with the specific dimensions would depend on the nature of these dimensions. We, however, had preliminary expectations. For example, we anticipated that fearful, authoritarian, and avoidant attitudes would relate to RWA, low openness to experience, low contact with people with MI, and disliking people with specific illnesses. Similarly, we expected that possible dimensions expressing lack of benevolence or sympathy would relate to SDO, low agreeableness, low empathy, limited contact, and disrespect for people with specific illnesses. We tested the measure across three studies, two employing select samples and the third involving participants from the general community.

## Study 1

### Method

#### Participants

The sample consisted of 301 participants (eight were removed as multivariate outliers), comprising university undergraduates (56.48%) and members of the general public. The mean age was 26.60 years (*SD* = 11.68), and respondents were predominantly female (78.45%) and Australian citizens (62.46%).

#### Materials and procedure

We recruited participants through predominantly psychology research websites and snowball sampling on social media. Although we provided online and paper versions of the survey, containing identical items, almost all participants completed the online version (98.3%). We measured the following constructs.

##### **Prejudice towards people with MI**

We conducted a thematic analysis [40] of the items in existing measures of attitudes and related constructs involving people with MI. Existing measures were identified based on a systematic review of the literature. The methodology employed in this review and the stages of the thematic analysis and reference measures are described in Additional file 1. We arranged items into 15 themes (see Additional file 1: Table S2). We decided to combine three themes due to content overlap, and to exclude six themes because they were not evaluations of people with MI. This left us with the seven remaining themes: dangerousness, unpredictability, authoritarianism, inferiority, social distance, interaction difficulty, and malevolence. We developed a pool of 179 items corresponding to the operational definitions of positively- and negatively-keyed items. Three experts rated items for their fidelity to the operational definitions and content validity. We selected the most highly rated items, paying attention to content overlap, to form a balanced 68-item scale with 8 or 10 items reflecting each theme. These were answered on a 9-point scale ranging from − 4 (*very strongly disagree*) to + 4 (*very strongly agree*).

##### **SDO and RWA**

Social dominance orientation was measured with a 16-item SDO scale [41] (α = .93), and RWA with the 18-item version of the Authoritarianism-Conservatism-Traditionalism (ACT) scale [42] (α = .88). Both used the same rating scale as the measure of prejudice above.

##### **Empathy**

We measured empathy using two 7-item subscales of the Interpersonal Reactivity Index [43]: empathic concern (α = .84) and perspective taking (α = .78).

##### **Social desirability**

We used a 10-item version (α = .66) of the Marlow-Crowne Social Desirability Scale [44].

##### **Big-five personality traits**

Participants completed a 50-item scale of personality traits from the International Personality Item Pool [45], including 10-item measures of extraversion, conscientiousness, neuroticism, agreeableness, and openness to experience (αs in this study ranged from .82 to .91). These, like measures of empathy and social desirability, were answered on a 5-point Likert scale ranging from 1 (*very inaccurate*) through to 5 (*very accurate*).

##### **Disliking and disrespect for people with specific MI**

Participants’ rated disliking and disrespect on 16 feeling thermometer scales related to people with depression, specific phobia, schizophrenia, obsessive-compulsive disorder, bipolar disorder, anxiety, eating, and substance use disorders. There were eight items measuring disliking and eight measuring disrespect, and the rating scale ranged from − 50 (*dislike* or *disrespect*) to + 50 (*like* or *respect*). Each measure was reverse-scored to indicate negativity. Exploratory factor analysis showed that there was one factor with an eigenvalue greater than 1 in both disliking (explaining 60.8% of variance) and disrespect items (73.4% of variance). Accordingly, we averaged participants’ scores and created measures of disliking (α = .91) and disrespect (α = .94).

##### **Discriminatory behaviour**

We developed a measure that asked participants how often (1 = *never*, 2 = *once*, 3 = *twice*, 4 = *three or more times*) they had engaged in six behaviours relating to people with MI (α = .70), based on a measure relating to gays/lesbians [46] (see Additional file 1).

##### **Contact**

Participants indicated *yes* or *no* to 12 items of the Level of Contact Report [47]. This measure assessed the level of past exposure of the respondent to people with a MI.

### Results and discussion

We imputed missing data (.19%) with expectation maximisation.

#### Exploratory factor analysis

We initially conducted confirmatory factor analysis (CFA),^1^ but failed to support the seven-factor model. Accordingly, data analysis moved into an exploratory phase: an exploratory factor analysis (EFA) using Principal Axis Factoring with Oblimin rotation. Eigenvalues, the scree plot and parallel analysis suggested six-factor and four-component solutions (see Additional file 1: Table S3). Although we explored the six-factor solution, we could not interpret it because there was a method factor (resulting from acquiescence) and one factor with two items. The four-factor solution (see Additional file 1: Table S4) was interpretable, demonstrating a simpler structure that explained 44.05% of the variance. Factor 1 explained 18.87% of the variance, and included social distance, dangerousness, and interaction difficulty items (named “fear/avoidance”). Factor 2 comprised inferiority and malevolence items and explained 12.72% of the variance (named “malevolence” given they both reflect unsympathetic attitudes). Factors 3 and 4 reflected the proposed dimensions of “authoritarianism” (14.84%) and “unpredictability” (9.75%). This supported our expectation about multidimensionality (Hypothesis 1).

It appears that even though the thematic analysis of prejudiced attitudes suggested that certain themes could be distinguishable conceptually, participants did not make such a distinction. Factor analysis is empirical and more objective than the more subjective thematic analysis. Our thematic analysis suggested that dangerousness, interaction difficulty, avoidance, malevolence, and inferiority appeared to be different constructs. Nonetheless, participants who perceived that people with MI are dangerous would also uniformly and automatically find that it is difficult to interact with them and that people with MI should be avoided. These three facets therefore formed one latent variable. In addition, participants who had malevolent attitudes towards people with MI also uniformly and automatically perceived them as inferior. Accordingly, these two facets formed one latent variable. Thus, instead of the hypothesised complex seven dimensions, the findings suggested a more parsimonious four-dimensional solution.

Based on factor analysis and item analysis we created a 28-item balanced scale: the Prejudice towards People with Mental Illness (PPMI) scale (α = .93), and four subscales, measuring fear/avoidance (α = .91), malevolence (α = .80), authoritarianism (α = .79), and unpredictability (α = .82). We selected items whose corrected item-to-.

subscale total correlation was above .3 and that loaded strongly onto the hypothesised factor, provided they did not load more strongly onto other factors. The scale demonstrated a readability (Flesch Reading Ease) score [48] of 60, suggesting its applicability to the general population. The dimensions were moderately to strongly intercorrelated, the strongest being between fear/avoidance and authoritarianism, *r* = .64, and the weakest between malevolence and unpredictability, *r* = .31.

#### Correlational analysis

Table 1 shows that prejudice towards people with MI as measured by the 28-item PPMI related to the proposed antecedents and consequences, supporting Hypotheses 2 and 3, and supporting the nomological network and convergent validity. More specifically, prejudice towards people with MI related: positively to SDO, RWA, disliking and disrespect for people with specific MI (Additional file 1: Table S5 includes specific correlations), and past discriminatory behaviours; and negatively to empathic concern, perspective taking, agreeableness, openness to experience, and contact. The scale did not significantly correlate with social desirability, demonstrating discriminant validity and absence of response bias.

**Table 1:** Correlations and semipartial correlations among PPMI scale and subscales, and hypothesised criterion variables (Study 1)

	PPMI	Fear/Avoidance		Malevolence		Authoritarianism		Unpredictability	
	*r*	*r*	*sr*	*r*	*sr*	*r*	*sr*	*r*	*sr*
SDO	.55**	.50***	.17***	.63***	.39***	.44***	.09*	.18***	−.14**
RWA	.51***	.40***	.02	.45***	.22***	.46***	.18***	.34***	.10*
Empathic concern	−.21***	−.19**	−.07	−.42***	−.37***	−.14*	.03	.07	.21***
Perspective taking	−.20***	−.18**	−.06	−.26***	−.14*	−.16**	−.03	−.07	.04
Extraversion	−.04	−.10	−.15**	−.09	−.07	.04	.13*	.05	.09
Agreeableness	−.18**	−.18***	−.01	−.32**	−.28***	−.10	.05	.05	.16**
Openness to experience	−.30***	−.22***	.02	−.25***	−.10	−.30***	−.16**	−.21***	−.06
Conscientiousness	−.04	−.03	.00	−.05	−.03	−.05	−.03	.00	.03
Neuroticism	.09	.06	−.01	.11	.09	.06	.00	.05	.02
Disliking PWSMI	.42***	.44***	.25***	.40***	.16**	.34***	.06	.14*	−.13**
Disrespect PWSMI	.38***	.39***	.21***	.38***	.19***	.28***	.02	.14*	−.10
Contact	−.37***	−.41***	−.28***	−.28***	−.07	−.24***	.05	−.22***	−.00
Social desirability	−.04	−.07	−.09	.01	.05	−.02	.01	−.02	.02
Past behaviour	.13*	.12*	.04	.20**	.15**	.11	.02	−.00	−.09

*N* = 301. PWSMI = People with Specific Mental Illnesses

* *p* < .05. ** *p* < .01. *** *p* < .001. The *p* values of the semipartial correlations are based on significance tests of the *B* coefficients obtained from the same regression analyses as the semipartial correlations

Our preliminary expectations about how the dimensions would relate to external variables appeared to be broadly supported. The semipartial correlations describing the unique associations of each subscale with external variables are shown in Table 1. First, fear/avoidance related negatively to contact, positively to disliking and disrespect for people with specific mental illnesses and, weakly, to SDO. Second, malevolence related positively to SDO and RWA, but negatively to empathic concern, perspective taking, agreeableness, and weakly positively with disliking and disrespect. Next, authoritarianism related positively to RWA and negatively to openness to experience. Finally, unpredictability related negatively to SDO, but positively to RWA, empathic concern, and agreeableness. No subscale related to social desirability. This pattern of findings and the differences in correlation sizes across subscales demonstrate convergent and discriminant validity. It should be noted that disliking and disrespect were strongly intercorrelated (*r* = .64, *p* < .001), suggesting that participants did not discriminate much between them. Interestingly, the dimensions of fear/avoidance and malevolence appeared to drive negative evaluations of people with specific MI.

As pointed out, one problem with measures in this area is the nonreplicable factor structure. In addition, in Study 1, we did not assess the relationship between the PPMI measure with an existing measure of attitudes towards people with MI and behaviours. Accordingly, we address these limitations and obtained further validity evidence in Study 2.

## Study 2

This study aimed to determine if the four-factor structure of the PPMI would be replicated in a second sample of participants. It also aimed to demonstrate concurrent validity of the PPMI scale using a widely-used measure of attitudes towards people with MI, the CAMI scale [14]. In addition, to further support the nomological network, we aimed to show that the subscales would predict behavioural intentions in situations eliciting relevant dimensions. For example, we hypothesised that fear/avoidance drives behaviours in situations where there is threat of contact with people with MI, malevolence drives behaviours related to disadvantaging people with MI, authoritarianism drives behaviours that involve controlling people with MI, and unpredictability drives unfavourable reactions to inconsistency of people with MI.

### Method

#### Participants

Participants were 164 undergraduate psychology students attending an Australian university (additional two were removed as multivariate outliers). There were 78.66% females, the mean age was 21.47 years (*SD* = 3.31), and most (75.61%) were Australians.

#### Materials and procedure

Participants completed a paper survey in a laboratory with the following measures.

##### **Prejudice towards people with MI**

The PPMI scale (α = .91) was included, with subscales reflecting fear/avoidance (α = .89), malevolence (α = .73), authoritarianism (α = .72), and unpredictability (α = .86). The scale included original 27 items from Study 1, but we substituted a somewhat weaker 28th item, which was, based on factor loadings, one of the weakest items to measure fear/avoidance (“I would not be comfortable having a neighbour who is mentally ill”) with a clearer item, which reflected the construct (“It is best to avoid people who have mental illness”). The original item also had the weakest factor loading and corrected item-total correlation in the final eight-item measure of all the selected items originally developed to measure dangerousness. Further, when the four factors were identified it became apparent that a clearer item reflecting the underlying construct was necessary to encapsulate the summarised construct of fear/avoidance. Following consultation with our expert panel of item raters, the decision was made to substitute a new item that more effectively reflected the fear/avoidance factor. Table 2 presents the items. The scale had an unchanged readability score (60). We also administered the CAMI scale ([14]; 40 items, α = .94). Both measures were answered on a 9-point scale ranging from − 4 (*very strongly disagree*) to + 4 (*very strongly agree*).

**Table 2:** Final 28 items from the PPMI scale and their factor loadings on the respective content factors from the CFA in Study 2 (*N* = 164) and 3 (*N* = 495)

Item	Study 2	Study 3
Fear/Avoidance		
I would find it hard to talk to someone who has a mental illness	.62	.63
I would be less likely to become romantically involved with someone if I knew they were mentally ill	.51	.45
It is best to avoid people who have mental illness	.76	.55
I would feel unsafe being around someone who is mentally ill	.81	.67
I would be just as happy to invite a person with mental illness into my home as I would anyone else*	.76	.77
I would feel relaxed if I had to talk to someone who was mentally ill*	.70	.75
I am not scared of people with mental illness*	.74	.68
In general, it is easy to interact with someone who has mental illness*	.67	.61
Malevolence		
People who are mentally ill are avoiding the difficulties of everyday life	.63	.35
People with mental illness should support themselves and not expect handouts	.58	.34
People who develop mental illness are genetically inferior to other people	.59	.45
People with mental illness do not deserve our sympathy	.49	.53
We, as a society, should be spending much more money on helping people with mental illness*	.48	.61
People who become mentally ill are not failures in life*	.41	.62
We need to support and care for people who become mentally ill*	.62	.75
Under certain circumstances, anyone can experience mental illness*	.42	.67
Authoritarianism		
People who are mentally ill need to be controlled by any means necessary	.67	.56
Those who have serious mental illness should not be allowed to have children	.57	.57
People who are mentally ill should be forced to have treatment	.49	.45
People who are mentally ill should be free to make their own decisions*	.51	.70
People who are mentally ill should be allowed to live their life any way they want*	.50	.66
Society does not have a right to limit the freedom of people with mental illness*	.39	.61
Unpredictability		
The behaviour of people with mental illness is unpredictable	.75	.74
People with mental illness often do unexpected things	.79	.75
In general, you cannot predict how people with mental illness will behave	.75	.77
The behaviour of people with mental illness is just as predictable as that of people who are mentally healthy*	.55	.47
People with mental illness behave in ways that are foreseeable*	.65	.25
I usually find people with mental illness to be consistent in their behaviour*	.67	.37

* = item was reverse-scored. All loadings were significant at *p* < 001

##### **Behavioural intentions scenarios**

We included a measure of behavioural intentions modelled on scenarios reflecting racial discrimination [49]. We asked participants to indicate their behaviours in five hypothetical scenarios, which reflected one of the four dimensions of prejudice towards people with MI: 1) interacting with someone who voiced the opinion that people with MI should not have children (authoritarianism would drive discriminatory behavioural intentions); 2) accepting that a coworker with MI had been passed over for promotion (malevolence should drive discrimination); 3) willingness to accept a person with MI being overlooked for a play due to possible unpredictability (unpredictability should drive discrimination); 4) willingness to live near a psychiatric institution (fear/avoidance should drive discrimination); and 5) tolerating a shopkeeper lying about a job’s availability to a person with MI (malevolence should drive discrimination). The full items are in Additional file 1.

### Results and discussion

We imputed missing data (1.29%) using expectation maximisation.

#### CFA

**Fig. 1:**
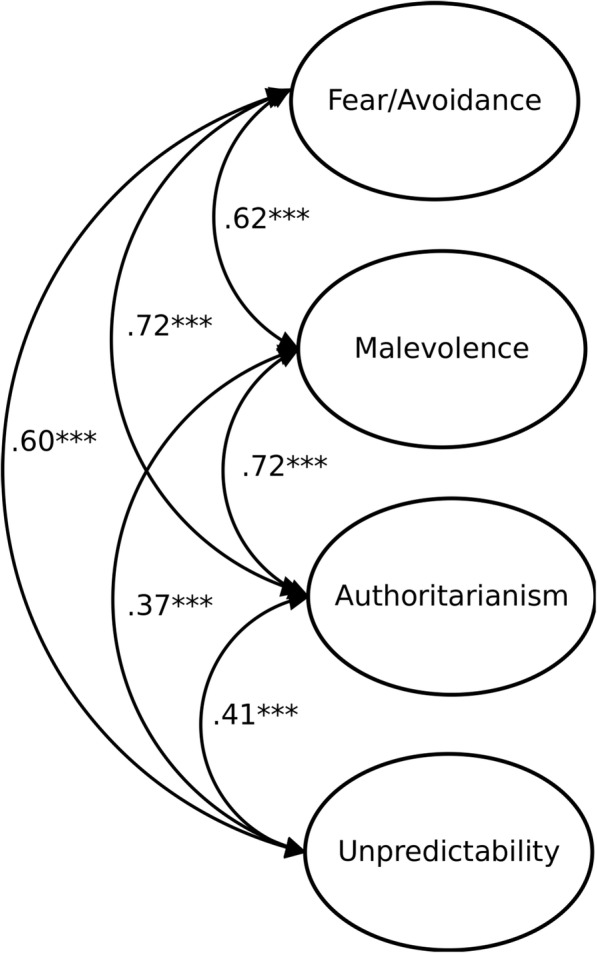
CFA of the four-factor model of Prejudice towards People with MI (Study 2). Manifest indicators are not shown. *N* = 164. *** *p* < .001

We analysed the variance-covariance matrix using maximum likelihood estimation. Given the moderate sample size, we used item parcels to reduce the number of parameter estimates [50],^2^ and control for acquiescence (we paired positively-keyed and negatively-keyed items in parcels). Guidelines [51] suggest that for acceptable model fit CFI should be larger than .95, and RMSEA and SRMR less than .08. The proposed four-factor model fit was acceptable, χ^2^(48) = 75.99, *p* = .006, CFI = .97, RMSEA = .06, SRMR = .04 (see Fig. 1), and better than that of alternative models: one-factor, χ^2^(54) = 353.13, *p* < .000, CFI = .68, RMSEA = .18, SRMR = .11, AIC = 425.13; and three-factor, which had items measuring fear/avoidance and authoritarianism loading on one factor, χ^2^(51) = 109.12, *p* < .001, CFI = .94, RMSEA = .08, SRMR = .06. Factor loadings of items from an item-level CFA on their respective four content factors are in Table 2.

#### Correlations

The PPMI scale and its subscales strongly correlated with the CAMI, demonstrating concurrent validity. The relationships ranged from *r* = .44 (unpredictability) to *r* = .78 (the overall scale).

### Ordinal logistic regression (OLR) analysis

We investigated the role of prejudice in behavioural intentions using OLR. As expected, fear/avoidance independently predicted unwillingness to rent an apartment near an inpatient facility, estimate = .34 [95% CI .01–.67], Wald χ^2^(1) = 4.52, *p* = .046. Malevolence predicted reluctance to speak out against a shopkeeper lying about a job’s availability to a person with MI, estimate = .58 [.09–1.07], Wald χ^2^(1) = 5.30, *p* = .021. Our expectation that authoritarianism would drive behavioural intentions in a scenario about prohibiting people with MI from having children was supported, estimate = .45 [.07–.82], Wald χ^2^(1) = 5.32, *p* = .021, although fear/avoidance was also a significant, though weaker, predictor, estimate = .41 [.03–.78], Wald χ^2^(1) = 4.52, *p* = .034. Contrary to hypotheses, malevolence, estimate = .84 [.16–1.53], Wald χ^2^(1) = 5.81, *p* = .016, but not unpredictability, drove discrimination in a scenario about rejecting people with MI from a role in a play, possibly because this discriminatory behaviour may disadvantage people with MI, and therefore may be elicited by malevolence. Also, contrary to predictions, malevolence did not predict lack of support for a mentally ill coworker’s promotion (no dimension was a significant predictor). Full results of OLR are in Additional file 1: Table S6.

The findings, therefore, partially supported the proposed nomological network of the PPMI subscales. We did not obtain evidence for the anticipated effects in every behavioural scenario, possibly because these were originally developed to measure racial discrimination and some of them required significant adaptation to be applicable for people with MI. Moreover, general attitudes best predict discrimination across situations, and not in one situation [52]. Overall findings, nonetheless, demonstrate the utility of the PPMI scale. The sample in this study, however, was specific and not large. Accordingly, we further tested the scale and its nomological network in the general population.

## Study 3

In this study, we aimed to further investigate the stability of the four-factor solution using item-level CFA in a large community sample consisting of participants from several countries. We also investigated the proposed nomological network of prejudice in relation to RWA, SDO, agreeableness, openness to experience, contact, and specific attitudes towards people with depression and schizophrenia. Finally, we also wanted to explore whether prejudice predisposes people to positive attitudes towards people who have never had MI.

## Method

### Participants

The sample consisted of 495 participants, who were recruited through the CrowdFlower service (a website that allows researchers to access community samples for a financial compensation). Participants were all native English-speakers (58.38% females, the mean age: 38.62, *SD* = 12.64). Data from non-native English-speakers and participants who speeded, engaged in response set, or failed attention checks were removed. Participants were citizens of the US (50.71%), the UK (21.21%), Canada (17.98%), and other countries (10.10%), with 76.77% self-identifying as Anglo/White.

### Materials and procedure

Participants completed an online survey with the following measures for a small payment.

#### **Prejudice towards people with MI**

We included the 28-item version of the PPMI scale (α = .91) from Study 2 (Table 2), with subscales measuring fear/avoidance (α = .87), malevolence (α = .83), authoritarianism (α = .82), and unpredictability (α = .79).

#### **SDO and RWA**

We included a newer 16-item SDO scale [33] (α = .93), and an 18-item version of the ACT scale [42] (α = .89). The SDO scale used a 7-point scale ranging from 1 (*strongly oppose*) to 7 (*strongly favor*). The ACT scale, like the PPMI scale, used a 9-point scale ranging from 1 (*very strongly disagree*) to 9 (*very strongly agree*).

#### **Big-five personality traits**

Participants completed 2-item measures of agreeableness and openness to experience from the balanced Ten Item Personality Inventory [53] using a 7-point scale ranging from 1 (*disagree strongly*) to 7 (*agree strongly*). The scales’ Spearman-Brown reliability coefficients were .47 for openness to experience and .57 for agreeableness.

#### **Contact**

Participants completed the 12-item Level of Contact Report as in Study 1.

#### **Feeling thermometer measure**

Participants indicated their level of favourability to people who have schizophrenia, depression, and those who have never had MI on a scale from − 50 (*unfavorable*) to + 50 (*favorable*).

## Results and discussion

### CFA

**Fig. 2:**
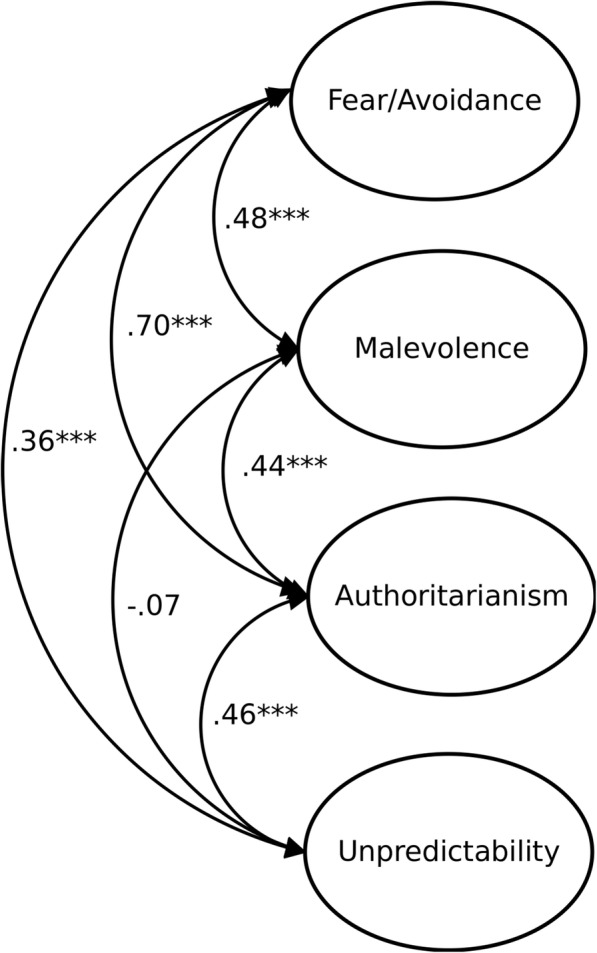
CFA of the four-factor model of Prejudice towards People with MI (Study 3). Manifest indicators and the uncorrelated method factors are not shown. *N* = 495. *** *p* < .001

We analysed the items’ variance-covariance matrix using maximum likelihood estimation. All tested models had content factors and two uncorrelated method factors, resulting from positively-keyed and negatively-keyed items, so that all positive items loaded on one and all negative on the other method factor; the method factors were not correlated with any other factor. This four-factor model, with additional two method factors, fitted the data well, χ^2^(316) = 870.21, *p* < .001, CFI = .92, RMSEA = .06, SRMR = .08. Apart from a non-significant correlation between malevolence and unpredictability, the four factors were moderately to strongly intercorrelated (see Fig. 2). A one-factor model, with two additional method factors, had a much worse fit to the data, χ^2^(322) = 1619.27, *p* < .001, CFI = .81, RMSEA = .09, SRMR = .14. Given the strongest intercorrelation between fear/avoidance and authoritarianism, we tested a three-factor model (also with two method factors) with items from these subscales loading on one factor. Its fit, χ^2^(319) = 1041.00, *p* < .001, CFI = .89, RMSEA = .07, SRMR = .12, was worse than the fit of the four-factor model. Accordingly, the analyses replicated the four-factor structure found in Studies 1 and 2. Factor loadings of items on their respective four content factors (see Table 2) tended to be similar to, though in several cases lower than, those in Study 2.

### Correlations

As in Study 1, and in support of Hypothesis 2 and 3, overall prejudice towards people with MI significantly correlated with hypothesised antecedents and consequence, supporting the nomological network (see Table 3): positively to SDO and RWA, negatively to agreeableness, openness to experience, contact, and specific attitudes towards people with schizophrenia and depression, but was unrelated to attitudes towards people who have never had MI. It should be noted that zero-order correlations of the PPMI subscales with other variables were as expected. Here, as in Study 1, we describe semipartial correlations. First, fear/avoidance related negatively to contact, agreeableness, openness to experience, and attitudes towards people with schizophrenia and depression. Second, malevolence related positively and very strongly to SDO, but more weakly to RWA; it also related negatively to agreeableness, openness to experience, attitudes towards people with schizophrenia and depression, and surprisingly to attitudes towards people who have never had MI (suggesting that malevolent prejudice may be part of a general misanthropic orientation, that is, negativity towards all humans). Next, authoritarianism related positively to RWA, and much more weakly to SDO and agreeableness. Finally, unpredictability related positively to RWA, agreeableness, and attitudes towards people who have never had MI, but negatively to attitudes towards people with schizophrenia. The overall pattern of findings is consistent with the results from Study 1, further supporting the nomological network and conceptual distinction between the dimensions.

**Table 3:** Correlations and semipartial correlations among PPMI scale and subscales, and hypothesised criterion variables (Study 3)

	PPMI	Fear/Avoidance		Malevolence		Authoritarianism		Unpredictability	
	*r*	*r*	*sr*	*r*	*sr*	*r*	*sr*	*r*	*sr*
SDO	.52**	.38***	−.003	.60***	.51***	.41***	.08*	.14**	.07
RWA	.39***	.24***	−.14***	.29***	.17***	.42***	.25***	.29***	.16***
Agreeableness	−.16**	−.20***	−.19***	−.23**	−.14**	−.05	.11*	.08	.11*
Openness to Experience	−.15***	−.16***	−.11*	−.19**	−.12**	−.08	.05	.01	.04
People Who Have Schizophrenia	−.43***	−.44***	−.25***	−.27***	−.08*	−.31***	.03	−.26***	−.10*
People Who Have Depression	−.44***	−.41***	−.17***	−.42***	−.24***	−.32***	−.01	−.13**	−.01
People Who Have Never Had MI	−.06	−.06	−.03	−.26***	−.23***	−.004	.05	.18***	.14**
Contact	−.20***	−.41***	−.26***	−.10*	.04	−.13**	.06	−.08	.03

*N* = 495. * *p* < .05. ** *p* < .01. *** *p* < .001. The *p* values of the semipartial correlations are based on significance tests of the *B* coefficients obtained from the same regression analyses as the semipartial correlations

### National and age differences

Given that Study 3 was more diverse in participants than the previous two studies we explored group and age differences. With respect to racial/ethnic groups, 76.77% were Anglo/Whites, and the other ethnic groups had a small number of participants (of the remaining groups the largest were Chinese with only 16 participants). Accordingly, we could not meaningfully compare Anglo/Whites with any other ethnic group. We, however, had three sufficiently large national groups for comparisons. A between-groups analysis of variance (ANOVA) including participants from the US, the UK, and Canada showed that there was a significant effect of nationality on overall prejudice to people with MI, *F*(2, 442) = 14.58, *p* < .001, η_p_^2^ = .06, fear/avoidance, *F*(2, 442) = 7.37, *p* < .001, η_p_^2^ = .03, authoritarianism, *F*(2, 442) = 8.71, *p* < .001, η_p_^2^ = .04, malevolence, *F*(2, 442) = 13.44, *p* < .001, η_p_^2^ = .06, and unpredictability, *F*(2, 442) = 6.04, *p* = .003, η_p_^2^ = .03.

Post-hoc comparisons using the Scheffe correction were then conducted. These revealed that on prejudice towards people with MI US participants, *M* = 4.30, *SD* = .97, were significantly higher than UK participants, *M* = 3.73, *SD* = 1.07, *t*(442) = 4.90, *p <* .001, and Canadian participants, *M* = 3.86, *SD* = 1.02, *t*(442) = 3.54, *p =* .002. US participants, *M* = 4.27, *SD* = 1.42, were also significantly higher on fear/avoidance than UK participants, *M* = 3.71, *SD* = 1.50, *t*(442) = 3.39, *p =* .003, and Canadian participants, *M* = 3.80, *SD* = 1.38, *t*(442) = 2.67, *p =* .029. Further, US participants, *M* = 4.39, *SD* = 1.47, were higher on authoritarianism than UK participants, *M* = 3.69, *SD* = 1.37, *t*(442) = 4.12, *p <* .001. US participants, *M* = 3.14, *SD* = 1.20, were also higher on malevolence than UK participants, *M* = 2.58, *SD* = 1.25, *t*(442) = 4.14, *p <* .001, and Canadian participants, *M* = 2.54, *SD* = 1.01, *t*(442) = 4.15, *p <* .001. Finally, US participants, *M* = 5.50, *SD* = 1.28, were higher on unpredictability than UK participants, *M* = 5.33, *SD* = 1.23, *t*(442) = 3.29, *p =* .005. No other national difference was significant.

There were no correlations of age with overall prejudice (*r* = −.05, *p* = .23), authoritarianism (*r* = .04, *p* = .35), and fear/avoidance (*r* = −.08, *p* = .07), but there was a significant negative correlation of age with malevolence (*r* = −.26, *p* < .001) and a significant positive correlation of age with unpredictability (*r* = .21, *p* < .001). This suggests that younger people tended to be more malevolent in their attitudes, and older perceived more unpredictability in people with MI.

## General discussion

To address limitations in the study of prejudice towards people with MI, we proposed and tested a new conceptualisation and nomological network of prejudice towards people with MI. Consistent with Hypothesis 1, prejudice was multidimensional. In contrast to our initial expectation, however, it consisted of four, and not seven, dimensions: fear/avoidance, malevolence, authoritarianism, and unpredictability. Next, supporting Hypothesis 2, prejudice related to the proposed antecedents: RWA and SDO (positively), agreeableness, openness to experience, empathy, and past contact with people with MI (negatively). This suggests that prejudice towards people with MI appears to be an outcome of ideology, personality, and past experiences. In fact, the strong associations with RWA and SDO indicated that ideology appears more important in predicting prejudice than the more widely studied contact with people with MI. Finally, supporting Hypothesis 3, prejudice predicted the proposed consequences: negative feelings for people with specific MI, past behaviours, and behavioural intentions. We demonstrated in this research that the scale measures the evaluative component of stigma, that is, prejudice, but that it is also consistently related to discrimination against people with MI. In addition, we showed that specific dimensions of prejudice lead to specific behavioural outcomes, enabling a nuanced understanding of the processes involved in stigma.

The PPMI scale demonstrated a consistent four-factor structure across three studies and in different cultural groups. This is significant as widely-used measures of attitudes towards people with MI, such as the OMI and CAMI, do not demonstrate a replicable factor structure. We demonstrated concurrent validity of the PPMI scale through correlating it with the CAMI scale [14]. This is an important finding because the PPMI scale is shorter and has improved psychometric properties.

Our findings support using both the subscales and the total scale. The model with four distinct factors was superior to the model with only one factor. Nevertheless, the four factors were intercorrelated and the overall scale was reliable, with Cronbach’s alphas in each study being above .90 (Study 1 α = .93, Study 2 α = .91, Study 3 α = .91). This suggests that there was significant internal consistency present in the items to sum them up also as a scale to measure prejudice as a multidimensional construct.

Using EFA and CFA, and by consistently demonstrating unique associations between each dimension and external variables, we provided strong evidence of multidimensionality and the nomological network across samples. These findings suggest that the four dimensions have distinct antecedents and consequences. For example, malevolence appears to be a function of low empathy, low agreeableness, and high SDO, and appears to strongly drive discriminatory behaviours.

This project has emphasised the importance of using theory and research to provide a strong conceptual foundation for a new measure. Our decision to focus on prejudice, as the component of stigma that influences behaviour, was driven by theoretical and practical considerations. By reviewing measures mainly from psychiatric and general population studies, we have encapsulated the aspects of prejudice targeted by stigma reduction campaigns and interventions. Positioning prejudice within a nomological network allowed us to demonstrate construct validity in a more comprehensive way than existing conceptualisations. It should be nevertheless noted that our initial expectation for seven dimensions was not supported, but once the four dimensions were discovered, they were repeatedly shown to exist across studies.

### Limitations and suggestions for future research

Our research has several limitations. First, we inferred the antecedents and consequences from theory and research, but we did not explicitly test whether ideological beliefs, personality, and contact cause prejudice towards people with MI. Although most theoretical approaches would assume that such factors would cause social attitudes [54], future experimental or longitudinal studies should address this issue. Next, whether it is useful to measure general attitudes towards people with MI can be questioned because there may be disparities between attitudes towards specific mental illnesses (e.g., [55, 56]). Nonetheless, Studies 1 and 3 show that general prejudice does correlate with feelings towards people with specific MI. Another limitation relates to the reliance on self-report to link prejudice with behaviour, and future research should use other methods, such as observing behaviour, to investigate the link. Finally, although we did not assess the PPMI scale for test-retest reliability, this scale was administered two weeks apart in another experiment [57], and partial correlations (controlling for the experimental condition) were satisfactory, suggesting good test-retest reliability: .73 (PPMI), .75 (fear/avoidance), .63 (malevolence), .71 (authoritarianism), and .63 (unpredictability).

By reviewing psychiatric and population research, we developed a measure that may prove useful in future research including intervention studies designed to reduce prejudice. The PPMI scale suggests that prejudice against people with MI consists of four main factors. Accordingly, anti-stigma interventions may target each of the specific attitudes separately and this may bring about more complex anti-stigma approaches. The measure could also be used to explore differences between interventions that have previously been obscured by unclear factor structures in existing measures. Indeed, our research shows that the current measure is useful in understanding nuanced changes in the four dimensions of prejudice following two interventions [57], but further investigation is needed.

## Conclusions

In this research, we clarified the structure of prejudice towards people with MI and positioned it in an empirically supported nomological network. We also presented evidence for an improved measure of prejudice, the PPMI scale. This research, therefore, provides a valuable theoretical and methodological contribution to the area, and fruitfully integrates approaches to stigma and prejudice. A novel contribution of this research is therefore the integration of stigma and prejudice literature in developing this measure, including its nomological network, and a very strong psychometric evaluation of the measure, encompassing a replicable factor structure, reliability, and validity.

Current intervention to reduce negative attitudes to people with MI are effective but the magnitude of the effects is small and there is a need to improve their effectiveness [58]. Such improvement must be informed by a better understanding of prejudice towards people with MI, including its antecedents and consequences, and by more reliable, comprehensive and precise measurement of it. The current research represents an important step towards achieving these goals.

## Additional file

Additional file 1: Stages of the Thematic Analysis of Items and Operational Definitions. **Table S1.** List of Measures Included in Thematic Analyses and Its Publication Reference. **Table S2.** Results of the Thematic Analysis. **Table S3.** Eigenvalues from Principal Axis Factoring and Simulated Eigenvalues from Parallel Analysis (Study 1). **Table S4.** Pattern Matrix for Principal Axis Factor Analysis with Oblimin Rotation of the PPMI Scale Items in Study 1. **Table S5.** Correlations of the PPMI Scale with Disliking or Disrespect for People with Specific Kinds of Mental Illness (Study 1). A Modified Measure of Past Behaviours Towards People with Mental Illness (Study 1). Modified Measures of Behavioural Intentions in Hypothetical Scenarios Involving People with MI (Study 2). **Table S6.** Ordinal Logistic Regression Analyses of Hypothetical Behaviour Scenarios Regressed on the PPMI Subscales (Study 2). (DOCX 42 kb)

## Abbreviations

ACT: Authoritarianism-conservatism-traditionalism
CAMI: Community attitudes toward the mentally ill
CFA: Confirmatory factor analysis
EFA: Exploratory factor analysis
MI: Mental illness
OLR: Ordinal logistic regression
OMI: Opinions about mental illness
PPMI: Prejudice towards people with mental illness
PWSMI: People with specific mental illnesses
RWA: Right-wing authoritarianism
SDO: Social dominance orientation

## Footnotes

1 The CFA for the seven-factor model did not demonstrate a clear solution, with modification indices suggesting that many items loaded strongly on two or more factors. Similarly, intercorrelations between factors were strong (up to .92, *p* < .001), indicating that several hypothesised factors measured the same construct.

2 We also tested an item-level CFA of the four-factor model with two additional method factors (i.e., positive and negative items loading on their respective method factors). It showed acceptable fit, χ^2^(316) = 551.32, *p* < .001, CFI = .88, RMSEA = .07, SRMR = .08, with all items loading on their hypothesised factors. The fit was better than that of alternative models, such as one-factor and three-factor models with method factors. This analysis was somewhat affected by the moderate sample size in relation to the model’s complexity.
