# Supplementary material for: The Prejudice towards People with Mental Illness (PPMI) scale: structure and validity
Source: BMC Psychiatry. 2018 Sep 12;18:293. doi: 10.1186/s12888-018-1871-z (PMC6142319; doi:10.1186/s12888-018-1871-z)
Supplement: Supplementary file 1 — Stages of the Thematic Analysis of Items and Operational Definitions. Table S1. List of Measures Included in Thematic Analyses and Its Publication Reference. Table S2. Results of the Thematic Analysis. Table S3. Eigenvalues from Principal Axis Factoring and Simulated Eigenvalues from Parallel Analysis (Study 1). Table S4. Pattern Matrix for Principal Axis Factor Analysis with Oblimin Rotation of the PPMI Scale Items in Study 1. Table S5. Correlations of the PPMI Scale with Disliking or Disrespect for People with Specific Kinds of Mental Illness (Study 1). A Modified Measure of Past Behaviours Towards People with Mental Illness (Study 1). Modified Measures of Behavioural Intentions in Hypothetical Scenarios Involving People with MI (Study 2). Table S6. Ordinal Logistic Regression Analyses of Hypothetical Behaviour Scenarios Regressed on the PPMI Subscales (Study 2). (DOCX 42 kb) [file 12888_2018_1871_MOESM1_ESM.docx]

**Supplementary Materials**

**Stages of the Thematic Analysis of Items and Operational Definitions**

Stage 1 – Compilation of measures

- Search terms in the PsycINFO database included “mental health”, “mental illness”, “mental disorder”, “stigma”, “attitude”, “instrument”, “measure”, and/or “scale”.
- Studies were located using the reference lists of other articles.
- Some measures overlapped with others, and some reflected specific attitudes/groups (e.g., attitudes towards schizophrenia or depression, attitudes of pharmacists, doctors, or nurses, and thus not included in the list of measures).
- The list of obtained references is in Table S1 in Supplementary Materials.
- Ideas for themes were noted during the process of familiarisation with measures.

Stage 2 – Identification of codes

- Each component of an item was given a unique code.
- Double-barrelled and complex items provided more than one code.
- We identified over 300 specific codes.
- Example code – DangO: People with mental illness are a danger to others.

Stage 3 – Arrangement into themes

- All similar codes were grouped together to form broad themes. This process required several iterations to resolve and incorporate all codes into overarching themes.
- The themes were checked against existing scales, literature on mental illness, and notes from Stage 1 to ensure coverage of the content breadth.

Stage 4 – Naming and defining the themes

- Fifteen themes were identified: dangerousness/fear, unpredictability, authoritarianism, capacity for independence, restrictiveness, inferiority, social distance, interaction difficulty, malevolence, causes of mental illness, knowledge of stigma, professionals’ perspectives, treatment efficacy (people), treatment efficacy (hospitals, medications), and stereotypes.
- Themes which did not reflect the definition of attitudes were excluded – causes of mental illness, knowledge of stigma, professionals’ perspectives, treatment efficacy (people), treatment efficacy (hospitals, medications), and stereotypes.
- In defining themes, it became clear that several were overlapping in content and were not conceptually distinct – therefore authoritarianism, capacity for independence, and restrictiveness were combined.
- Thus, seven themes were identified and hypothesised to reflect attitudes towards people with mental illness. These were dangerousness, unpredictability, authoritarianism, inferiority, social distance, interaction difficulty, and malevolence.
- Operational definitions of positively-keyed and negatively-keyed items were created.
  - Dangerousness
    - A belief that people with mental illness are dangerous.
    - A belief that people with mental illness are harmless.
  - Unpredictability
    - A belief that the behaviour of people with mental illness is unpredictable.
    - A belief that people with mental illness behave predictably.
  - Authoritarianism
    - A belief that people with mental illness should be controlled by others by any means necessary.
    - A belief that people with mental illness should be free to control their own lives.
  - Inferiority
    - A belief that people with mental illness are inferior to other people.
    - A belief that people with mental illness are the same as other people.
  - Social distance
    - A belief that people with mental illness should be avoided and rejected from the community.
    - A belief that with mental illness should be accepted and integrated into the community.
  - Interaction difficulty
    - A belief that people with mental illness are difficult to interact with.
    - A belief that people with mental illness are easy to interact with.
  - Malevolence
    - A belief that people with mental illness do not deserve any sympathy or support.
    - A belief that people with mental illness deserve sympathy and support.

Table S1. List of Measures Included in Thematic Analyses and Its Publication Reference

| Measure and Reference |
| --- |
| Opinions about Mental Illness (OMI) [1] |
| Custodial Mental Illness Ideology scale (CMI) [2] |
| Community Mental Health Ideology scale (CMHI) [3] |
| Community Attitudes Toward the Mentally Ill (CAMI) [4] |
| Social Distance/Perceived Dangerousness Questionnaires [5] |
| Devaluation-Discrimination Questionnaire [6] |
| Beliefs toward Mental Illness scale (BMI) [7] |
| Stigmatisation of people with mental illness [8] |
| Mental Health Consumer Participation Questionnaire [9] |
| Medical Condition Regard Scale (MCRS) [10] |
| Attribution Questionnaire (AQ) [11] |
| Mental Illness Disorder and Understanding Scale (MIDUS) [12] |
| Mental Disorder Prejudice Scale (MDPS) [13] |
| Attitudes Toward Serious Mental Illness Scale – Adolescent Version (ATSMI-AV) [14] |
| Attitudes Towards Acute Mental Health Scale (ATAMHS) [15] |
| Standardised Stigmatisation Questionnaire 1 (SSQ1) [16] |
| Attitudes to Mental Illness Questionnaire (AMIQ) [17] |
| Personal/Perceived Stigma Scale [18] |
| Mental Illness Stigma Scale [19] |
| Stigma Questionnaire (SQ) [20] |
| Consumer Participation and Consultant Questionnaire (CPCQ) [21] |
| Stigmatising Attitudes-Believability Scale (SAB) [22] |
| HealthStyles Survey [23] |
| Beliefs and attitudes towards people with mental problems [24] |
| Mental Health Knowledge Schedule (MAKS) [25] |
| Mental Illness: Clinicians’ Attitudes (MICA) [26] |
| Fear and Behavioural Intentions towards the mentally ill (FABI) [27] |

**References**

1. Cohen J, Struening EL. Opinions about mental illness: Mental hospital occupational profiles and profile clusters. Psychological Reports. 1963;12:111–24.

2. Gilbert DC, Levinson DJ. Ideology, personality, and institutional policy in the mental hospital. In: Smelser NJ, Smelser WT, editors. Personality and social systems. Hoboken, NJ: Wiley; 1963. p. 619–29.

3. Baker F, Schulberg HC. The development of a Community Mental Health Ideology scale. Community Mental Health Journal. 1967;3:216–25.

4. Taylor SM, Dear MJ. Scaling community attitudes toward the mentally ill. Schizophrenia Bulletin. 1981;7:225–40.

5. Link BG, Cullen FT, Frank J, Wozniak JF. The social rejection of former mental patients: Understanding why labels matter. American Journal of Sociology. 1987;92:1461–500.

6. Link BG. Understanding labeling effects in the area of mental disorders: An assessment of the effects of expectations of rejection. American Sociological Review. 1987;52:96–112.

7. Hirai M, Clum GA. Development, reliability, and validity of the Beliefs toward Mental Illness scale. Journal of Psychopathology and Behavioral Assessment. 2000;22:221–36.

8. Crisp AH, Gelder MG, Rix S, Meltzer HI, Rowlands OJ. Stigmatisation of people with mental illnesses. British Journal of Psychiatry. 2000;177:4–7.

9. Happell B, Pinikahana J, Roper C. Attitudes of postgraduate nursing students towards consumer participation in mental health services and the role of the consumer academic. International Journal of Mental Health Nursing. 2002;11:240–50.

10. Christison GW, Haviland MG, Riggs ML. The Medical Condition Regard Scale: Measuring reactions to diagnoses. Academic Medicine. 2002;77:257–62.

11. Corrigan PW, Rowan D, Green A, Lundin R, River P, Uphoff-Wasowski K, et al. Challenging Two Mental Illness Stigmas: Personal Responsibility and Dangerousness. Schizophr Bull. 2002;28:293–309.

12. Tanaka G, Ogawa T, Inadomi H, Kikuchi Y, Ohta Y. Effects of an educational program on public attitudes towards mental illness. Psychiatry and Clinical Neurosciences. 2003;57:595–602.

13. Tanaka G, Inadomi H, Kikuchi Y, Ohta Y. Evaluating stigma against mental disorder and related factors. Psychiatry and Clinical Neurosciences. 2004;58:558–66.

14. Watson AC, Miller FE, Lyons JS. Adolescent attitudes toward serious mental illness. Journal of Nervous and Mental Disease. 2005;193:769–72.

15. Baker JA, Richards DA, Campbell M. Nursing attitudes towards acute mental health care: Development of a measurement tool. Journal of Advanced Nursing. 2005;49:522–9.

16. Haghighat R. The development of an instrument to measure stigmatization: Factor analysis and origin of stigmatization. European Journal of Psychiatry. 2005;19:144–54.

17. Luty J, Fekadu D, Umoh O, Gallagher J. Validation of a short instrument to measure stigmatised attitudes towards mental illness. Psychiatric Bulletin. 2006;30:257–60.

18. Griffiths KM, Nakane Y, Christensen H, Yoshioka K, Jorm AF, Nakane H. Stigma in response to mental disorders: A comparison of Australia and Japan. BMC Psychiatry. 2006;6:21.

19. Day EN, Edgren K, Eshleman A. Measuring stigma toward mental illness: Development and application of the Mental Illness Stigma scale. Journal of Applied Social Psychology. 2007;37:2191–219.

20. Littlewood R, Jadhav S, Ryder AG. A cross-national study of the stigmatization of severe psychiatric illness: Historical review, methodological considerations and development of the questionnaire. Transcult Psychiatry. 2007;44:171–202.

21. McCann TV, Clark E, Baird J, Lu S. Mental health clinicians’ attitudes about consumer and consumer consultant participation in Australia: A cross-sectional survey design. Nursing and Health Sciences. 2008;10:78–84.

22. Masuda A, Price M, Anderson PL, Schmertz SK, Calamaras MR. The role of psychological flexibility in mental health stigma and psychological distress for the stigmatizer. Journal of Social and Clinical Psychology. 2009;28:1244–62.

23. Kobau R, DiIorio C, Chapman D, Delvecchio P, Members SMISP. Attitudes about mental illness and its treatment: Validation of a generic scale for public health surveillance of mental illness associated stigma. Community Ment Health J. 2010;46:164–76.

24. Aromaa E, Tolvanen A, Tuulari J, Wahlbeck K. Attitudes towards people with mental disorders: the psychometric characteristics of a Finnish questionnaire. Soc Psychiat Epidemiol. 2010;45:265–73.

25. Evans-Lacko S, Little K, Meltzer H, Rose D, Rhydderch D, Henderson C, et al. Development and psychometric properties of the mental health knowledge schedule. Can J Psychiatry. 2010;55:440–8.

26. Kassam A, Glozier N, Leese M, Henderson C, Thornicroft G. Development and responsiveness of a scale to measure clinicians’ attitudes to people with mental illness (medical student version). Acta Psychiatrica Scandinavica. 2010;122:153–61.

27. Svensson B, Markström U, Bejerholm U, Björkman T, Brunt D, Eklund M, et al. Test - retest reliability of two instruments for measuring public attitudes towards persons with mental illness. BMC Psychiatry. 2011;11:11.

Table S2. Results of the Thematic Analysis

| Theme Number | Theme Name |
| --- | --- |
| 1 | Dangerousness/fear |
| 2 | Unpredictability |
| 3 | Authoritarianism |
| 4 | Capacity for independence |
| 5 | Restrictiveness |
| 6 | Inferiority |
| 7 | Social distance |
| 8 | Interaction difficulty |
| 9 | Malevolence |
| 10 | Causes of mental illness |
| 11 | Knowledge of stigma |
| 12 | Professionals’ perspectives |
| 13 | Treatment efficacy (people) |
| 14 | Treatment efficacy (hospitals, medications) |
| 15 | Stereotypes |

*Legend.* Themes 3-5 were subsequently merged because they appeared not to be sufficiently conceptually distinct from each other. Themes 10-15 did not reflect the definition of attitudes towards people with MI and were excluded.

Table S3. Eigenvalues from Principal Axis Factoring and Simulated Eigenvalues from Parallel Analysis (Study 1)

| Factor | PAF | PA |
| --- | --- | --- |
| 1 | 23.252 | 1.323 |
| 2 | 3.586 | 1.230 |
| 3 | 2.013 | 1.156 |
| 4 | 1.494 | 1.094 |
| 5 | 1.323 | 1.044 |
| 6 | 1.079 | 0.996 |
| 7 | 0.919 | 0.943 |
| 8 | 0.890 | 0.901 |
| 9 | 0.740 | 0.861 |
| 10 | 0.709 | 0.824 |

*Legend.* *N* = 301.

Table S4. Pattern Matrix for Principal Axis Factor Analysis with Oblimin Rotation of the PPMI Scale Items in Study 1

|  | Fear/Avoidance | Malevolence | Unpredictability | Authoritarianism |
| --- | --- | --- | --- | --- |
| I would find it hard to talk to someone who has a mental illness ** | .89 |  |  |  |
| In general, it is easy to interact with someone who has mental illness ** - *R* | .81 |  |  |  |
| I would feel relaxed if I had to talk to someone who was mentally ill ** - *R* | .80 |  |  |  |
| I tend to feel uneasy when I am with someone who has a mental illness | .74 |  |  |  |
| I don’t think I can relax and be myself when I’m around someone with a mental illness | .69 |  |  |  |
| I feel as comfortable talking to a person with mental illness as I do talking to a person with physical illness - *R* | .68 |  |  |  |
| I am not scared of people with mental illness ** - *R* | .67 |  |  |  |
| I would be just as happy to invite a person with mental illness into my home as I would anyone else ** - *R* | .65 |  |  |  |
| People with mental illness are easy to talk to - *R* | .62 |  |  |  |
| Having a chat with someone who is mentally ill would not be difficult- *R* | .61 |  |  |  |
| I would feel comfortable having a person with mental illness marry into my family - *R* | .59 |  |  |  |
| I would be less likely to become romantically involved with someone if I knew they were mentally ill ** | .52 |  |  |  |
| I would not mind if a person with mental illness took care of my children - *R* | .52 |  |  |  |
| I think a person relationship with someone who has a mental illness would be too demanding | .45 |  |  |  |
| I would not be comfortable having a neighbour who is mentally ill ** | .43 |  |  |  |
| I would feel unsafe being around someone who is mentally ill ** | .43 |  |  |  |
| It is safe to be around people who are mentally ill - *R* | .42 |  |  | -.40 |
| I believe that it is acceptable to have someone with mental illness as an employee - *R* | .41 |  |  |  |
| It is not good to become friends with a person who has a mental illness | .41 | .31 |  |  |
| When I am around someone who has a mental illness I am worried they may harm me physically | .37 |  |  | -.30 |
| People with mental illness should be integrated in the community - *R* | .36 |  |  | -.31 |
| Mental health treatment facilities should be kept out of residential neighbourhoods | .35 |  |  |  |
| The public does not need protection from people who are mentally ill - *R* | .34 |  |  |  |
| A close relationship with someone with a mental illness would be like living on an emotional rollercoaster | .32 |  | .36 |  |
| People with mental health problems are often dangerous |  |  | .36 |  |
| I think people with mental illness often pose a risk to other people |  |  | .34 |  |
| A person with mental illness is more likely to harm others than a mentally healthy person |  |  |  | -.35 |
| People have nothing to fear from people with mental illness - *R* |  |  |  | -.31 |
| People who have mental illness are just as likely to be violent as the mentally healthy - *R* |  |  |  |  |
| We need to support and care for people who become mentally ill ** - *R* |  | .78 |  |  |
| We, as a society, should be spending much more money on helping people with mental illness ** - *R* |  | .63 |  |  |
| We should do as much as we can to help those who suffer from mental illness - *R* |  | .63 |  |  |
| People with mental illness deserve much more than what we are giving them now - *R* |  | .63 |  |  |
| People with mental illness should support themselves and not expect handouts ** |  | .61 |  |  |

|  | Fear/Avoidance | Malevolence | Unpredictability | Authoritarianism |
| --- | --- | --- | --- | --- |
| People with mental illness do not deserve our sympathy ** |  | .56 |  |  |
| Our taxes should not go toward people who are mentally ill |  | .52 |  |  |
| People who become mentally ill lack self-discipline more than others |  | .46 |  | -.30 |
| People with mental illness are not in any way inferior to others - *R* |  | .44 |  |  |
| People who become mentally ill are not failures in life ** - *R* |  | .43 |  |  |
| People who are mentally ill are avoiding the difficulties of everyday life ** |  | .43 |  |  |
| Under certain circumstances, anyone can experience mental illness ** - *R* |  | .42 |  |  |
| People who develop mental illness are genetically inferior to other people ** |  | .40 |  |  |
| Mental illness occurs in people with a weak personality |  | .39 |  | -.35 |
| Society should not feel responsible for people who become mentally ill |  | .38 |  |  |
| People with mental illness need support from other people - *R* |  | .37 |  |  |
| People with mental illness should be locked away |  | .36 |  | -.38 |
| I would not mind if all the mentally ill people were expelled from my country |  | .34 |  | -.30 |
| People with mental illness are a huge burden on society | .34 | .31 |  |  |
| People with mental illness often do unexpected things ** |  |  | .74 |  |
| In general, you cannot predict how people with mental illness will behave ** |  |  | .70 |  |
| The behaviour of people with mental illness is unpredictable ** |  |  | .66 |  |
| You cannot tell what people with mental illness will do from one minute to the next |  |  | .53 |  |
| The behaviour of people with mental illness is just as predictable as that of people who are mentally healthy ** - *R* |  |  | .51 |  |
| People with mental illness behave in ways that are foreseeable ** - *R* |  |  | .49 |  |
| I usually find people with mental illness to be consistent in their behaviour ** - *R* |  |  | .44 |  |
| You always know what to expect from someone who has a mental illness - *R* |  |  | .38 |  |
| Mental illness is as normal as physical illness - *R* |  |  |  | -.32 |
| People with mental illness typically have the same level of intelligence as the general public - *R* |  |  |  | -.31 |
| Mentally ill people are in many ways like children |  |  |  |  |
| People who are mentally ill should be free to make their own decisions ** - *R* |  |  |  | -.71 |
| People who are mentally ill should be allowed to live their life any way they want ** - *R* |  |  |  | -.62 |
| Just because someone is mentally ill does not mean others should control them - *R* |  |  |  | -.57 |
| People who are mentally ill need to be controlled by any means necessary ** |  |  |  | -.49 |
| Society does not have a right to limit the freedom of people with mental illness ** - *R* |  |  |  | -.43 |
| People who are mentally ill should be forced to have treatment ** |  |  | .35 | -.40 |
| People who are mentally ill should not be denied any rights - *R* |  |  |  | -.39 |
| Those who have serious mental illness should not be allowed to have children ** |  |  |  | -.37 |
| People with mental illness require control and discipline |  |  | .42 | -.31 |

*Legend*. *N* = 301. Item loadings greater than .30 are reported. ** Items included in the Study 1 28-item four-factor scale. *R* = reverse scored

Table S5. Correlations of the PPMI Scale with Disliking or Disrespect for People with Specific Kinds of Mental Illness (Study 1)

|  | PPMI and Disliking  *r* | PPMI and Disrespect  *r* |
| --- | --- | --- |
| Specific phobia | .17** | .23*** |
| Depression | .31*** | .31*** |
| Schizophrenia | .49*** | .39*** |
| Obsessive-compulsive disorder | .26*** | .32*** |
| Bipolar disorder | .48*** | .39*** |
| Eating disorder | .27*** | .35*** |
| Substance use disorder | .39*** | .32*** |

*Legend.* *N* = 301*.* ** *p* < .01. *** *p* < .001.

**A Modified Measure of Past Behaviours Towards People with Mental Illness (Study 1)**

I have laughed at jokes about people with mental illness.

I have described a person with mental illness using a term such as “psycho” or “demented”.

I have avoided someone because they looked mentally ill.

I have ended a friendship with someone when I learned they were mentally ill.

I have made derogatory statements about people with mental illness.

I have threatened a mentally ill person with violence.

**Modified Measures of Behavioural Intentions in Hypothetical Scenarios Involving People with MI (Study 1)**

1. Imagine that you are in class and your instructor has broken the class into small groups to discuss the rights of people with mental illness. One of the students in your group says it would be great if people with mental illness had more rights in society but they shouldn’t be allowed to have children. Under these conditions,

a) I would voice my disagreement with the student.

b) I would disagree with the student but not say anything.

c) I would agree with the student but not say anything.

d) I would voice my agreement with my student.

2. Imagine that one of your co-workers has a mental illness. You notice that they tend to get the worst job assignments and have been passed over for promotion although they seemed to deserve it. Under these conditions,

a) I would go the next higher supervisor and tell her or him what’s going on.

b) I would express my concerns to my mentally ill co-worker.

c) I wouldn’t want to create problems, so I would probably stay out of the situation.

d) I would feel that the supervisor knows what’s right.

3. Imagine you are a member of a casting committee for a drama club that is in the process of casting parts for a new play. The casting committee is in complete agreement about the actor who deserves the lead role. One committee member mentions that this actor has a mental illness and has been seeing a psychiatrist for a while. As a result of this, the rest of the committee decides that he should probably not get the part. Under these conditions,

a) I would say that if they refuse to give the part to the best qualified actor I would resign from the committee.

b) I would say that the actor should be judged on his talent not his mental illness; but I would go along with any decision the majority made.

c) I wouldn’t know what to do so I’d go along with whatever the majority wanted.

d) I would agree that it was not a good idea to cast someone who is mentally ill.

4. Imagine you are looking for an apartment to rent that you saw advertised in the paper. You stop a stranger who is watering his lawn to ask for directions. The person you have stopped gives you the directions but says, “You don’t want to live there, that place is full of loonies because it’s near a mental institution”. Under these conditions,

a) I would tell him living near a mental institution didn’t make any difference to me.

b) I would be offended by his comment, but I wouldn’t say anything.

c) I wouldn’t respond to his comment, but if he was right I probably wouldn’t rent it.

d) I would thank him for his advice and I would no longer consider living in that apartment building.

5. Imagine you are in a store waiting to make a purchase. Across the aisle, a man asks the manager about a sales position that is open, and is given an application to complete and return. Several minutes later another man approaches the manager about the same job opening. He tells the manager that there are some gaps in his resume due to periods of mental illness, however now he is fully recovered. The manager tells him the position has already been filled. Under these conditions,

a) I would confront the manager about his discriminatory actions.

b) I would make my purchase and would probably write a letter of complaint to the manager.

c) I would stay out of it because it wouldn’t make much different to me one way or the other.

d) I would feel it is the right of the management to not employ mentally ill people if they want.

Table S6. Ordinal Logistic Regression Analyses of Hypothetical Behaviour Scenarios Regressed on the PPMI Subscales (Study 2)

| Scenario (hypothesised PPMI subscale) | PPMI Subscale | Estimate | Error | Wald test | Significance | Confidence interval (of estimate) |
| --- | --- | --- | --- | --- | --- | --- |
| No children (authoritarianism) | **Fear/avoidance** | **.41** | **.19** | **4.52** | **.034** | **.03-.78** |
|  | Malevolence | -.16 | .27 | .36 | .549 | -.69-.37 |
|  | **Authoritarianism** | **.45** | **.19** | **5.32** | **.021** | **.07-.82** |
|  | Unpredictability | .08 | .17 | .25 | .620 | -.24-.41 |
| Co-worker promotion (malevolence) | Fear/avoidance | .27 | .17 | 2.54 | .111 | .06-.59 |
|  | Malevolence | .20 | .24 | .72 | .396 | -.26-.66 |
|  | Authoritarianism | .04 | .17 | .04 | .835 | -.20-.37 |
|  | Unpredictability | -.17 | .14 | 1.64 | .201 | -.44-.09 |
| Casting in a play (unpredictability) | Fear/avoidance | .16 | .22 | .52 | .472 | -.30-.58 |
|  | **Malevolence** | **.84** | **.35** | **5.81** | **.016** | **.16-1.53** |
|  | Authoritarianism | .04 | .22 | .03 | .869 | -.40-.47 |
|  | Unpredictability | .21 | .17 | 1.63 | .202 | -.11-.54 |
| Renting apartment (fear/avoidance) | **Fear/avoidance** | **.34** | **.17** | **3.97** | **.046** | **.01-.67** |
|  | Malevolence | .38 | .24 | 2.47 | .116 | -.10-.86 |
|  | Authoritarianism | -.14 | .17 | .67 | .412 | -.48-.20 |
|  | Unpredictability | -.03 | .14 | .05 | .819 | -.30-.24 |
| Shopkeeper lying (malevolence) | Fear/avoidance | .21 | .17 | 1.48 | .224 | -.13-.54 |
|  | **Malevolence** | **.58** | **.25** | **5.3** | **.021** | **.09-1.07** |
|  | Authoritarianism | -.17 | .18 | .96 | .326 | -.52-.17 |
|  | Unpredictability | -.10 | .14 | .57 | .451 | -.37-.17 |

*Legend.* *N* = 164. Statistically significant findings are in bold.
